# Caution

**Published:** 1875-11

**Authors:** 


### CAUTION.

We hear of some imitations of Hall's Journal of Health in process of preparation. This thing of issuing bogus Journals under our name, or what the law terms and punishes as a "colorable variation" of our title, has been practised by some dishonest persons for several years, and a good deal of money has been lost by those who have been deceived into subscribing for journals which come out perhaps once or twice a year, after promising to appear monthly. The publisher of the genuine Hall's Journal of Health,—now closing its twenty-second year,—is the American and Foreign Publication Company, and its office is 137 East Eighth Street, New York. Letters thus addressed will reach the right source, and money thus sent will be duly accounted for. If not so sent, we can not be held responsible.

